# Work-related injuries of rehabilitation therapists and measures for prevention

**DOI:** 10.3389/fpubh.2024.1398948

**Published:** 2024-07-31

**Authors:** Wei Liu, Weibo Tian, Tianyu Wu, Bin Luo, Jiang Yi, Wenmao Li, Junjie Jiang, Yanlong Wei, Tianqi Zhang, Fengyue Zhang, Xiaoqin Duan, Bin Zheng

**Affiliations:** ^1^Department of Rehabilitation Medicine, Jilin University Second Hospital, Changchun, China; ^2^Department of Neurology and Neuroscience Center, The First Hospital of Jilin University, Changchun, China; ^3^Sanya Rehabilitation and Convalescent Center, Joint Logistics Support Force, Sanya, China; ^4^Department of Rehabilitation Medicine, Second People’s Hospital of Beihai, Beihai, China; ^5^Surgical Simulation Research Lab, Department of Surgery, University of Alberta, Edmonton, AB, Canada

**Keywords:** work-related injuries, rehabilitation therapists, prevention, questionnaire survey, rehabilitation medicine

## Abstract

**Background:**

Rehabilitation therapists often perform physically demanding treatments that may result in work-related injuries, yet related studies are scarce. This study aimed to investigate the work-related injuries of rehabilitation therapists and provide feasible preventive measures.

**Methods:**

A cross-sectional study was carried out in 34 regions across China using an online questionnaire. The survey gathered responses primarily from 1,198 rehabilitative therapists working in 120 health institutes. Following data collection, descriptive analysis, chi-square tests, logistic regression, and receiver operating characteristic (ROC) curves were employed to analyze the data.

**Results:**

In this study, the incidence of work-related injuries was reported to be 87% (*n* = 1,041). The top three musculoskeletal issues reported were low back pain (12%), neck pain (10%), and shoulder pain (9%). Logistic regression and ROC curve analysis identified that working as a physiotherapist and years of work experience (OR [95% CI]: 1.03 [0.99–1.07]) were significant contributors to the incidence of work-related injuries. Specifically, neuro-physiotherapists (OR [95% CI]: 3.04 [1.56–5.92]), musculoskeletal physiotherapists (OR [95% CI]: 2.46 [1.16–5.18]), and intensive care physiotherapists (OR [95% CI]: 4.70 [1.24–17.88]) were at higher risk. Furthermore, five factors were proven to be associated with injury prevention as reported by therapists: patient engagement (OR [95% CI]: 0.38 [0.23–0.62]), improving techniques (OR [95% CI]: 0.59 [0.39–0.90]), maintaining exercise habits (OR [95% CI]: 0.59 [0.40–0.86]), utilizing instruments (OR [95% CI]: 0.80 [0.53–1.19]), and strengthening education (OR [95% CI]: 0.43 [0.21–0.90]).

**Conclusion:**

The present study investigated the factors contributing to work-related injuries among rehabilitation therapists, with a focus on identifying both risk and preventive measures. These findings offer new perspectives on decreasing injury risk.

## 1 Introduction

Rehabilitation therapists provide specialized treatments focused on enhancing the movement and functionality of individuals with various conditions such as hemiplegia, paraplegia, quadriplegia, and other related disorders. These interventions typically involve a combination of manipulative techniques and therapeutic exercises aiming at improving the overall quality of life for patients. However, it is important to note that physical therapists, the most common type of rehabilitation therapists, are at a higher risk of experiencing work-related injuries (1, 2). The increasing incidence of work-related injuries among physical therapists has resulted in a significant attrition rate within the profession, with many practitioners exiting the field within a few years of practice (3).

Musculoskeletal disorders and injuries are considered to be the most common health problems in the contemporary labor force (4). Studies have shown that physical therapists experience various work-related injuries. Milhem et al. conducted a study that revealed the occurrence of musculoskeletal injuries, with lower back issues being the most common at 45%, followed by wrist or hand (29.6%), upper back (28.7%), and neck (24.7%) disorders (5). Another study highlighted that hand therapists often experience work-related injuries or pain concentrated around the metacarpophalangeal or carpometacarpal thumb joints (6). Additionally, physical therapists specializing in acute care, geriatrics, and pediatrics commonly experience lower back problems, whereas those specializing in orthopedics and neurology are more prone to neck issues (7).

However, most previous studies were conducted within a single institution, focusing on incidents instead of assessment of related factors. Warren et al. identified three primary paradigms as the most influential work risk models for assessing work-related risks, as reported by individuals themselves. These paradigms included “performing the same task repeatedly,” “working in the same position for prolonged periods,” and “treating a large number of patients within a single day” (8). Recent studies have expanded on this by identifying specific work practices that increased the risk of therapist-related low back pain (LBP), such as lifting weights, transporting patients, and performing repetitive movements (5). However, existing studies have been limited by their narrow focus on specific work types and locations, thereby leaving the global risk factors for occupational injuries among rehabilitation therapists largely unexplored. Additionally, there is a notable lack of studies that addressing measures to mitigate discomfort or injuries related to occupational hazards.

Therefore, the aim of this cross-sectional study was to explore the incident of work-related injuries and the potential risk and preventive factors measures for work-related injuries among rehabilitation therapists. We surveyed various therapists at multiple institutions using an online questionnaire, including physical therapists, occupational therapists, speech therapists, traditional chiropractors, acupuncturists, and others, to gather information about work-related injuries. The self-reported data were used to develop new preventive strategies for work-related injuries.

## 2 Methods

### 2.1 Study design

A cross-sectional survey was conducted in 34 regions in China in April of 2022. The questionnaire (attached in the appendix) consisted of twenty-one questions encompassing various aspects, such as basic information, workload, time of work, type, and severity of disorders or pain, potential causes, and preventive measures. The present survey was designed to gather self-reported data on work-related factors among physical therapists (PTs), which followed the STROBE guidelines for comprehension from this cross-sectional investigation (9). Additionally, the initial questions were selected based on established instruments used in previous studies (10–12). Before sending the questionnaire online, 10 PTs from academic, hospital, and private medication institutions additionally reviewed each question and evaluated the survey to ensure the validity and clarity of the questionnaire. Of note, the questionnaires were only distributed to prospective participants who were registered physiotherapists in over 120 medical organizations. At the same time, therapists who were retired or not actively employed at the time of the survey were excluded.

### 2.2 Data collection

The survey was sent to over 120 health institutes through the “Questionnaire Star,” a secured online survey App (Ranxing Information Technology Co., Ltd., Changsha, China) in Chinese. Questionnaires and survey methods used in the study were reviewed and approved by the Ethics Review Board of the Second Hospital of Jilin University (No. 2022-047). The consent page was displayed to each participant at the beginning of the online survey; the survey started when a participant clicked on the “Agree” button on the consent page.

### 2.3 Questionnaire content

In this questionnaire, the risk factors associated with work-related injuries were derived from two parts: (1) Personal Characteristics: This section of the questionnaire gathered participants’ demographics, lifestyle, and work-related information, including age, gender, height, weight, education, and leisure physical activity. Work-related questions comprised years of professional experience, working hours per week in the main physical therapy job, and number of patients treated per week. (2) Occupation-Related Issues: For participants who reported occupation-related discomforts, the questionnaire included an additional section listing potential reasons and preventive measures for the discomfort, as well as the distribution and degree of the injuries.

### 2.4 Statistical analysis

Descriptive statistics were used for analyzing the incidence of discomfort, injuries, and preventive measures reported by participants. To determine the risk factors associated with work-related injuries, we performed Chi-square tests to analyze differences among data gathered from therapists with self-reported injuries and therapists without self-reported injuries. A multivariate logistic regression model was constructed using the factors that were considered clinically important or that showed a possible univariate connection (*p* value <0.2) with work-related injuries. Finally, we plotted ROC curves to estimate risk factors of work-related injuries and preventive measures. All data were statistically analyzed and plotted using SPSS20.0 (IBM Corporation, Armonk, New York, United States) and R software 4.3.1.^1^ Results were reported by mean ± standard deviation; *p* < 0.05 was considered to be statistically significant.

## 3 Results

### 3.1 Demographics

Between April 19, 2022, and April 22, 2022, we received a total of 1,198 completed questionnaires from 34 regions in China. 1,041 therapists (87%) reported experiencing discomfort or injuries due to their work with patients. This survey includes various types of therapists such as neuro-physiotherapists, musculoskeletal physiotherapists, occupational therapists, speech therapists, physiotherapists for intensive care units, traditional chiropractors, acupuncturists, and others. All therapists were actively involved in clinical treatment and regularly engaged in manual work that required prolonged physical exertion and energy. Their responses to the questionnaire were based on their professional experience. The summary of participant characteristics is described in Table 1. Two sections were included in the demographical survey: (1) personal characteristics: age, gender, BMI, exercise habits, and relevant education on prevention for work-related injuries. (2) Work-related issues: working time (years), time of work per week (days), time of work per day (hours), frequency of treatment of patients per day (*n*), work unit types, and preventive measure of work-related injuries. Among the 1,041 therapists who reported discomforts or injuries in this survey, 473 were males, and 568 were females, while there were 80 men and 75 women among 155 participants reported no discomfort or injury. The main occupational types were neuro-physiotherapist, musculoskeletal physiotherapist, and Occupational therapist. The average BMI were 25.3 and 24.6 kg/m^2^ among therapists with or without injuries, respectively.

**Table 1:** General information and comparisons of therapists.

Variables		Therapist with worked-related injuries (*n* = 1,041)	Therapist without worked-related injuries (*n* = 155)	*p*
**Age (years)**		30.57 ± 6.32	30.51 ± 8.05	0.946
**Gender (*n*)**				0.176
Male		473 (45.4%)	80 (51.6%)	
Female		568 (54.6%)	75 (48.4%)	
**BMI (kg/m** ^**2**^ **)**		25.3 ± 10.0	24.6 ± 6.97	0.308
**Exercise**				0.001**
Have a habit of regular exercise		36% (371)	50% (78)	
Do not have a habit of regular exercise		64% (670)	50% (77)	
**Relevant educational pathways**	School curriculum	8% (83)	14% (21)	0.024*
	Peer communication	39% (409)	53% (83)	0.001**
	Self-study	31% (324)	22% (34)	0.020*
	Never studied	22% (225)	11% (17)	0.002**
**Working time (years)**		7.26 ± 5.10	6.46 ± 6.25	0.143
**Time of work per week (days)**		5.49 ± 0.63	5.50 ± 0.63	0.822
**Time of work per day (hours)**		7.59 ± 1.07	7.55 ± 1.26	0.716
**Frequency of treatment of patients per day (*n*)**		11.69 ± 9.67	10.59 ± 6.58	0.081
**Type of occupation**	Therapist working for physical factor therapy	6% (66)	12% (18)	0.016*
	Neuro-physiotherapist	40% (420)	32% (49)	0.039*
	Musculoskeletal physiotherapist	18% (191)	15% (23)	0.278
	Occupational therapist	12% (121)	15% (24)	0.168
	Speech therapist	7% (68)	8% (12)	0.572
	Intensive physiotherapist	5% (50)	2% (3)	0.106
	Traditional chiropractor	2% (26)	4% (6)	0.322
	Acupuncturist	3% (32)	5% (7)	0.344
	Others	6% (67)	8% (13)	0.363
**Work Unit**	The tertiary general hospitals	67% (696)	61% (94)	0.132
	The secondary general hospitals	18% (186)	19% (30)	0.650
	The community health organizations	2% (20)	4% (6)	0.120
	Specialized rehabilitation organizations	11% (115)	10% (16)	0.764
	Health-related industry organizations	2% (24)	6% (9)	0.013*
**Protective measures at work**	Changing posture	29% (922)	24% (130)	0.122
	Utilizing equipment	17% (528)	18% (98)	0.005**
	Improving technology	15% (483)	18% (99)	<0.001***
	Wearing protective device	8% (270)	7% (39)	0.914
	Patient engagement	20% (643)	23% (127)	<0.001***
	Making full use of interns and trainees	9% (305)	9% (50)	0.510
	No protective measures	2% (54)	2% (9)	0.897

Intergroup comparison: *p < 0.05, **p < 0.01, ***p < 0.001.

### 3.2 Problems and precautions

Table 2 reported the common injuries-related issues among 1,041 therapists with work-related injuries. The most commonly reported discomforts or injuries among 1,041 therapists included low back pain (12%), neck pain (10%), and shoulder pain (9%). For the degree of pain, over half of the participants reported experiencing significant pain levels. The majority of responses indicated that these discomforts or injuries typically manifest within 1 to 3 years of starting their profession. Self-reports identified frequent manipulation (39%) and poor posture (35%) as the primary causes of pain. Additionally, among the 1,041 therapists who reported injuries, they also suggested various measures to alleviate symptoms, 24% chose to strengthen self-exercise, 22% chose to rest and allow their bodies to recover, 21% sought rehabilitation treatment from their colleagues, 17% preferred to adopt labor-saving or protective positions during work (Table 2).

**Table 2:** The general work-related issues among the therapist with worked-related injuries.

Issues among therapist with worked-related injuries (*n* = 1,041)	*N*	%
**Pain types**		
Neck pain	810	11.7%
Shoulder pain	711	10.3%
Elbow pain	263	3.8%
Wrist pain	568	8.2%
Hand pain	474	6.8%
Chest and back pain	424	6.1%
Low back pain	920	13.3%
Hip pain	282	4.1%
Knee pain	316	4.6%
Ankle pain	155	2.2%
Foot pain	207	3.0%
Numbness in forearm or finger	259	3.7%
Tired easily	583	8.4%
Headache insomnia	273	3.9%
Chest distress and shortness of breath	530	7.7%
Others	147	2.1%
**The degree of pain**		
No pain	9	0.9%
Mild pain	466	44.8%
Significant pain that they could tolerate	505	48.5%
Had trouble sleeping due to pain	55	5.3%
Extreme pain	6	0.6%
**Years of discomfort**		
Within 1 year	229	22.0%
1–3 years	453	43.5%
3–6 years	263	25.3%
6–9 years	68	6.5%
More than 9 years	28	2.7%
**Potential causes of injuries**		
Poor posture	9	0.9%
Frequent manipulation	466	44.8%
Technique flaw	505	48.5%
Natural degeneration	55	5.3%
Psychological stress	6	0.6%

### 3.3 Comparisons

A comparison between therapists with and without work-related injuries revealed several noteworthy findings. When we grouped all participants based on their associated health institutes, we found that participants working at all types of institutes reported experiencing injuries and without injuries (Tertiary: 67% vs. 61%; Secondary: 18% vs. 19%; Specialized: 11% vs. 10%); however, only in those health-related industry organization, participants experienced more non-injuries than those with injuries (6% vs. 2%, *p* < 0.05). The proportion of therapists who engaged in regular exercise in the non-work-related injury group was significantly higher than that in the work-related injury group (*p* < 0.01). Furthermore, a higher percentage of therapists in the non-work-related injury group received protective education through school curriculum (*p* < 0.05) or peer communication (*p* < 0.01) compared to that in the work-related injury group. The proportion of therapists in the injury group receiving protective education through self-study (*p* < 0.05) or not receiving protection education (*p* < 0.01) was higher than that of therapists in the non-work-related injury group. Moreover, the percentage of therapists in the non-work-related injury group adopting posture change as a protective measure was lower than that of the work-related injury group (*p* < 0.05). A higher proportion of therapists working for physical factor therapy in the non-work-related injury group compared to that of the work-related injury group (*p* < 0.05). Lastly, the percentage of therapists working as neuro-physiotherapists in the injury group was higher than that in the non-work-related injury group (*p* < 0.05).

Both groups displayed relatively similar patterns in terms of the therapists’ work profiles, with the top three occupations being neuro-physiotherapist (40 and 32%), musculoskeletal physiotherapist (18 and 15%), and occupational therapist (12 and 15%). In both groups, therapists received relevant protection education through peer communication (39 and 53%), self-study (31 and 22%), and school education (8 and 14%). The recommended order of protective measures for both groups aligned, including changing posture (24 and 29%), patient engagement (23 and 20%), utilizing equipment (18 and 17%), improving techniques (18 and 15%), fully utilizing interns and trainees (9 and 9%), and wearing protective device (7 and 8%). Notably, the proportion of individuals without regular exercise habits was higher in the work-related injury group compared to that of individuals with regular exercise habits (*p* < 0.01) (Table 1).

### 3.4 Diagnostic ability of various factors for work-related injuries

To identify potential risk factors and preventive factors for work-related injuries of physical therapists, we conducted logistic regression between various traits reported and the outcome of injuries (Table 3). The results indicated specific occupation types positively associated with increased work-related injuries, including neuro-physiotherapists (OR [95%CI]: 3.04 [1.56–5.92], *p* < 0.01), musculoskeletal physiotherapists (OR [95%CI]: 2.46 [1.16–5.18], *p* < 0.01), and intensive physiotherapists (OR [95%CI]: 4.70 [1.24–17.88], *p* < 0.05).

**Table 3:** The results of multivariable logistic regression.

Characteristic	Beta	S.E	OR(95%CI)	*p*
**Working time (years)**	0.03	0.02	1.03 (0.99–1.07)	0.114
**Type of occupation**				
Therapist working for physical factor therapy	Ref	Ref	1	1
neuro-physiotherapists	1.11	0.34	3.04 (1.56–5.92)	0.001**
musculoskeletal physiotherapists	0.9	0.38	2.46 (1.16–5.18)	0.018*
occupational therapists	0.52	0.38	1.68 (0.80–3.55)	0.172
speech therapists	0.36	0.46	1.44 (0.59–3.51)	0.423
physiotherapists for intensive care unit	1.55	0.68	4.70 (1.24–17.88)	0.023*
traditional chiropractors	0.62	0.61	1.86 (0.57–6.10)	0.307
acupuncturists	−0.06	0.55	0.94 (0.32–2.79)	0.914
other therapists	0.62	0.46	1.85 (0.75–4.59)	0.183
**Work unit**				
The tertiary general hospitals	Ref	Ref	1	1
The secondary general hospitals	0.69	0.49	1.99 (0.76–5.25)	0.164
The community health organizations	0.65	0.52	1.91 (0.69–5.34)	0.216
Specialized rehabilitation organizations	−0.09	0.69	0.91 (0.24–3.50)	0.894
Health-related industry organizations	0.75	0.56	2.12 (0.71–6.40)	0.180
**Exercise**				
Do not have a habit of regular exercise	Ref	Ref	1	1
Have a habit of regular exercise	−0.53	0.19	0.59 (0.40–0.86)	0.006**
**Protective measures at work**				
Utilizing equipment	−0.23	0.21	0.80 (0.53–1.19)	0.271
Improving technology	−0.52	0.21	0.59 (0.39–0.90)	0.014*
Patient engagement	−0.96	0.25	0.38 (0.23–0.62)	<0.001***
Changing posture	0.82	0.27	2.27 (1.33–3.88)	0.103
**Relevant educational pathways**				
Never	Ref	Ref	1	1
Self-study	−0.03	0.33	0.97 (0.50–1.86)	0.919
Peer communication	−0.63	0.29	0.53 (0.30–0.95)	0.032*
School curriculum	−0.84	0.38	0.43 (0.21–0.90)	0.026*

*p < 0.05, **p < 0.01, ***p < 0.001.

**Figure 1:**
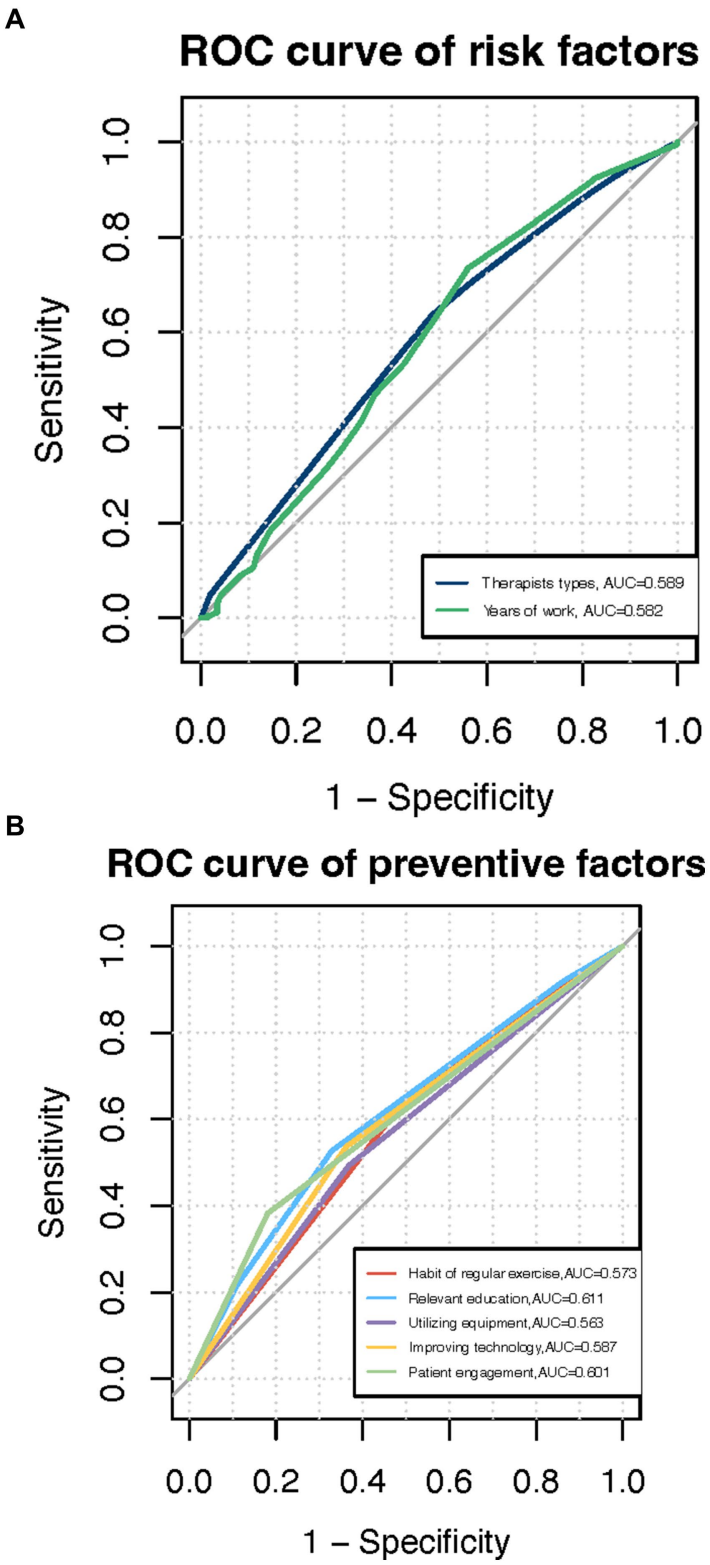
**(A)** ROC curves of various factors for work-related injuries, **(B)** ROC curves of prevention strategies for work-related injuries.

Furthermore, the ROC curves were created to assess the diagnostic capability of various factors in predicting work-related injuries. To assess the diagnostic capability of various factors in predicting work-related injuries, we created ROC curves. The areas under the curve (AUC) of two specific factors, namely work as PTs (neuro-physiotherapists, musculoskeletal physiotherapists, and intensive physiotherapists) and years of work were greater than 0.5 (*p* < 0.05) (Figure 1A).

### 3.5 Prevention values of various factors for work-related injuries

As in Table 3, the results of logistic regression indicated that regular exercise (OR [95%CI]: 0.59 [0.40–0.86], *p* < 0.01) and education about work-related injuries (OR [95%CI]: 0.43 [0.21–0.90], *p* < 0.05) could be the potential preventive factors. Additionally, two interventive measures were found to be associated with decreased work-related injuries, including patient engagement (OR [95%CI]: 0.38 [0.23–0.62], *p* < 0.001), and improving techniques (OR [95%CI]: 0.59 [0.39–0.90], *p* < 0.05).

Consistently, the ROC curves assessed the preventive value of different factors for work-related injuries. To assess the preventive value of different factors for work-related injuries, we generated ROC curves. As shown in Figure 1B, there were five factors demonstrating preventive effects for work-related injuries, including patient engagement (AUC = 0.601, *p* < 0.01), improving techniques (AUC = 0.587, *p* < 0.01), regular exercise habits (AUC = 0.574, *p* < 0.01), utilizing equipment (AUC = 0.562, *p* < 0.05) and receiving relevant education for work-related injuries (AUC = 0.551, *p* < 0.05) (Figure 1B).

## 4 Discussion

We conducted a comprehensive survey encompassing a significant number of therapists from various regions in China, who are directly involved in patient care. The survey extensively addressed general information, work-related injuries, protective measures, and their educational experiences in this field. The main findings of the present study suggested several work-related factors associated with rehabilitation therapists.

Work-related musculoskeletal disorders (WMSD) refer to musculoskeletal issues that are either caused or worsened by work activities. They are the main type of work-related injuries and are the fastest growing contributor to work-related disability (9). Early detection and intervention of WMSD based on occupational risk factors have been shown to enhance the overall prognosis (10). Physical therapists (PTs) are susceptible to WMSD due to the nature of their daily tasks, which often involve repetitive movements, prolonged postures, and heavy lifting (11). It has been reported that approximately 1 in 6 PTs had to modify their workload or even exit the profession due to WMSD (12). This study conducted a cross-sectional survey among PTs from China using a questionnaire, which initially identified various types of work-related injuries associated with their profession. In the present study, we identified initially the types of self-reported WMSD that occurred in PTs. The top three discomforts reported by them were low back pain, neck pain, and shoulder pain, accounting for the highest incident, which was consistent with previous studies. Hence, there is a growing emphasis on the importance of proactively addressing and preventing work-related injuries at an early stage.

Previous studies have reported several risk factors associated with work-related injuries in therapists, including age, gender, high workload, body mass index, lack of professional experience, working in private clinics, and working in a seated position (13, 14). However, these studies were all exclusively conducted in Europe. To be noted, Campo’s research highlighted that physiotherapists and occupational therapists were prone to experiencing high rates of work-related pain (15). Similarly, a recent study also confirmed the high risk of WMSD among PTs compared to the general population (16). In line with these findings, the results of our survey consistently suggested that working as physiotherapists (neuro-physiotherapists, musculoskeletal physiotherapists, intensive physiotherapists), as well as the number of years of work were potential diagnostic factors for work-related injuries in China. These findings support that different types of occupational roles carry varying risks of work-related injuries, suggesting the need for precise occupational protection for special PTs, especially neuro-physiotherapists, musculoskeletal physiotherapists, and intensive physiotherapists. Interestingly, we did not find any association between gender and injury risk, warranting further investigation through longitudinal cohort studies.

Furthermore, our survey revealed that poor posture (35%) and frequent manipulation (39%) were identified as the primary causes of discomfort among the participating therapists. This aligns with the nature of their work. Physiotherapists often engage in direct patient training, which can involve prolonged periods of restricted postures, repetitive tasks, application of high-speed forces, and movements that require bending and twisting (17). Similarly, Ezzatvar et al. that poor posture and high workload were associated with incident of work-related injuries in physical therapists (13). Physiotherapists may treat a substantial number of patients within a single day and even continue to work while injured, contributing to the increased risk of injuries (5, 14, 18). Working in a standing position places mechanical stress on the spine, potentially leading to spinal problems among physiotherapists (16). Therefore, it is necessary to establish healthy posture and therapy range guidelines for therapists, especially for PTs working for a long time, which may help lower the risk of work-related injuries.

To reduce the occurrence of work-related injuries, therapists employ various protective measures. Interestingly, our findings indicate that a higher proportion of therapists who reported injuries opted to change their posture compared to those who did not report any injuries. This suggests that simply changing posture may not be highly effective in preventing work-related injuries. In our study, we recommend employing strategies such as equipment utilization, technique improvement, and patient engagement to prevent work-related injuries. Among these strategies, instrument-assisted soft tissue mobilization (IASTM) stands out as a valuable approach that utilizes rehabilitative tools to efficiently detect and treat soft tissue pathologies, which provides therapists with a more effective treatment option (17). Patient engagement, which involves shifting some or all of the physical workload to another person, can be an effective measurement for preventing injuries of therapists (17). To be noted, regular exercise was supported as a precise protective factor against WMSD in the present study, which was consistent with the study by Ludewig. Their study has indicated effective protective values of the home exercise program on shoulder pain in construction workers (19). These findings suggest several preventive measures for occupational injuries among physical therapists, advocating for a comprehensive strategy that integrates a conducive work environment with regular physical activity in daily routines to help reduce the likelihood of such injuries.

Among the various factors that contribute to the prevention of occupational injuries, education plays a crucial role. Specifically, it is essential to offer physiotherapists instruction in ergonomic and biomechanical principles, along with practical training in patient care, in order to reduce the incidence of work-related injuries (20). Insufficient ergonomic training for physical therapists may increase their susceptibility to workplace injuries (21). In this study, the effectiveness of preventing work-related injuries was demonstrated through peer communication, self-study, and school education. The findings indicate that purely theoretical instruction aimed at improving human mechanics for injury prevention is less effective than experiential teaching (3). Consequently, it is important to increase practical education for PTs to reduce the incidence of work-related injuries in the future.

This study has some limitations. There are more than 60,000 rehabilitation therapists in China at least. However, the number of therapists surveyed in this questionnaire was just over 1,000, which may not provide a comprehensive representation of the work-related injuries of all therapists. Moreover, the assessment of risk and preventive factors was primarily done by logistic regression and ROC curve, which may not fully capture all relationships. Additionally, this is a cross-sectional study with exposure and outcome assessed simultaneously. To validate the relationships between risk and preventive factors among PTs, prospective cohort studies are essential to conduct.

## 5 Conclusion

The results of this study suggest that several work-related factors among PTs. Specific occupation types of PTs (especially neuro-physiotherapists, musculoskeletal physiotherapists, and intensive physiotherapists) and years of work were identified as risk factors for PTs. To prevent such injuries, therapists should be encouraged to use equipment, actively involve patients in their treatment, and maintain regular exercise. This was particularly crucial for therapists who had been practicing for extended periods. Hospitals and health institutes should take measures to increase technical training for therapists and provide comprehensive education programs on injury prevention. The findings of this study may be taken into account for establishing clinical guidelines, interventions, and improved working circumstances for physical therapists.

## Data availability statement

The original contributions presented in the study are included in the article/Supplementary material, further inquiries can be directed to the corresponding authors.

## Ethics statement

The studies involving humans were approved by Research Ethics Board of the Second Hospital of Jilin University. The studies were conducted in accordance with the local legislation and institutional requirements. The participants provided their written informed consent to participate in this study.

## Author contributions

WeiL: Data curation, Methodology, Software, Visualization, Writing – original draft. WT: Data curation, Visualization, Writing – original draft, Writing – review & editing. TW: Investigation, Resources, Writing – original draft. BL: Data curation, Writing – review & editing. JY: Data curation, Writing – review & editing. WenL: Data curation, Writing – review & editing. JJ: Data curation, Writing – review & editing. YW: Data curation, Writing – review & editing. TZ: Data curation, Writing – review & editing. FZ: Data curation, Writing – review & editing. XD: Conceptualization, Resources, Writing – review & editing, Methodology, Visualization. BZ: Supervision, Writing – review & editing.

## Footnotes

^1^ https://www.r-project.org/
