# Supplementary material for: Work-related injuries of rehabilitation therapists and measures for prevention
Source: Front Public Health. 2024 Jul 31;12:1398948. doi: 10.3389/fpubh.2024.1398948 (PMC11322811; doi:10.3389/fpubh.2024.1398948)
Supplement: Supplementary file 1 [file Table_1.DOCX]

Supplementary Material

Work-related Injuries of Rehabilitation Therapists and Measures for Prevention

**Wei Liu**^1^†, **Weibo Tian**^1,2^†, **Tianyu Wu**^3^, **Bin Luo**^4^, **Jiang Yi**^1^, **Wenmao Li**^1^, **Junjie Jiang**^1^, **Yanlong Wei**^1^, **Tianqi Zhang**^1^, **Fengyue Zhang**^1^, **Xiaoqin Duan**^1^*, **Bin Zheng**^5^*

*** Correspondence:** Xiaoqin Duan^*^, MD, Ph.D., [15204309769@163.com;](mailto:15204309769@163.com;) Bin Zheng^*^,[bin.zheng@ualberta.ca.](mailto:bin.zheng@ualberta.ca;)

**Lable:**

**Table 1:** The results of univariable logistic regression

**Table 2:** Results of ROC curves of various factors for work-related injuries.

**Table 3:** Results of ROC curves of prevention strategies for work-related injuries.

**Table 1: The results of univariable logistic regression**

| **Characteristic** | **N(%)** | **OR (95%CI)** | **P** |
| --- | --- | --- | --- |
| **Age** | 30.6 (6.57) | 1.00 (0.97 - 1.03) | 0.935 |
| **Gender** | | | |
| Male | 553 (46.2%) | 1 | 1 |
| Female | 643 (53.8%) | 1.28 (0.91 - 1.80) | 0.151 |
| **BMI (kg/m2)** | 25.2 (9.69) | 1.01 (0.99 - 1.03) | 0.427 |
| **Working time (years)** | 7.16 (5.27) | 1.03 (1.00 - 1.07) | 0.087 |
| **Time of work per week (days)** | 5.49 (0.63) | 0.97 (0.74 - 1.28) | 0.823 |
| **Time of work per day (hours)** | 7.59 (1.10) | 1.03 (0.88 - 1.21) | 0.682 |
| **Frequency of treatment of patients per day (n)** | 11.5 (9.34) | 1.02 (0.99 - 1.04) | 0.189 |
| **Type of occupation** | | | |
| Therapist working for physical factor therapy | 84 (7.02%) | 1 | 1 |
| neuro-physiotherapists | 469 (39.2%) | 2.338 (1.284 - 4.257) | 0.005 ** |
| musculoskeletal physiotherapists | 214 (17.9%) | 2.265 (1.150 - 4.458) | 0.018 * |
| occupational therapists | 145 (12.1%) | 1.375 (0.696 - 2.716) | 0.359 |
| speech therapists | 80 (6.69%) | 1.545 (0.691 - 3.457) | 0.289 |
| physiotherapists for intensive care unit | 53 (4.43%) | 4.545 (1.269 - 16.288) | 0.020 * |
| traditional chiropractors | 32 (2.68%) | 1.182 (0.422 - 3.308) | 0.750 |
| acupuncturists | 39 (3.26%) | 1.247 (0.473 - 3.288) | 0.656 |
| other therapists | 80 (6.69%) | 1.406 (0.638 - 3.098) | 0.398 |
| **Degree** | | | |
| junior college | 193 (16.1%) | 1 | 1 |
| bachelor | 905 (75.7%) | 1.21 (0.78 - 1.90) | 0.396 |
| Master | 81 (6.77%) | 0.89 (0.43 - 1.82) | 0.744 |
| Doctor | 9 (0.75%) | 0.59 (0.12 - 3.01) | 0.529 |
| Others | 8 (0.67%) | 1.19 (0.14 - 10.03) | 0.874 |
| **Protective measures at work** | | | |
| Changing posture |  |  |  |
| No | 144 (12.0%) | 1 | 1 |
| Yes | 1052 (88.0% | 1.49 (0.93 - 2.38) | 0.095 |
| Utilizing equipment |  |  |  |
| No | 570 (47.7%) | 1 | 1 |
| Yes | 626 (52.3%) | 0.60 (0.42 - 0.85) | 0.004** |
| Improving technology |  |  |  |
| No | 614 (51.3%) | 1 | 1 |
| Yes | 582 (48.7%) | 0.49 (0.35 - 0.69) | <0.001*** |
| Wearing protective device |  |  |  |
| No | 887 (74.2%) | 1 | 1 |
| Yes | 309 (25.8%) | 1.04 (0.71 - 1.54) | 0.837 |
| Patient engagement |  |  |  |
| No | 426 (35.6%) | 1 | 1 |
| Yes | 770 (64.4%) | 0.36 (0.23 - 0.55) | <0.001*** |
| Making full use of interns and trainees |  |  |  |
| No | 841 (70.3%) | 1 | 1 |
| Yes | 355 (29.7%) | 0.87 (0.61 - 1.25) | 0.452 |
| No protective measures |  |  |  |
| No | 1133 (94.7% | 1 | 1 |
| Yes | 63 (5.27%) | 0.89 (0.43 - 1.84) | 0.748 |
| **Relevant educational pathways** | | | |
| Never | 242 (20.2%) | 1 | 1 |
| Self-study | 358 (29.9%) | 0.98 (0.51 - 1.88) | 0.947 |
| Peer communication | 492 (41.1%) | 0.54 (0.30 - 0.96) | 0.036** |
| School curriculum | 104 (8.70%) | 0.43 (0.21 - 0.90) | 0.025** |
| **Exercise** | | | |
| Don't have a habit of regular exercise | 747 (62.5%) | 1 | 1 |
| Have a habit of regular exercise | 449 (37.5%) | 0.58 (0.40 - 0.84) | 0.004** |
| **Work Unit** | | | |
| The tertiary general hospitals | 790 (66.1%) | 1 | 1 |
| The secondary general hospitals | 216 (18.1%) | 0.84 (0.54 - 1.30) | 0.431 |
| The community health organizations | 26 (2.17%) | 0.45 (0.18 - 1.15) | 0.095 |
| Specialized rehabilitation organizations | 131 (11.0%) | 0.97 (0.55 - 1.71) | 0.918 |
| Health-related industry organizations | 33 (2.76%) | 0.36 (0.16 - 0.80) | 0.012* |

Note: *P<0.05, **P<0.01***, P<0.001.

**Table 2:** Results of ROC curves of various factors for work-relted injuries

| **Area Under Curve (AUC)** | | | | | |
| --- | --- | --- | --- | --- | --- |
| Test variable | AUC | standant error^a^ | Progressive Sig.^b^ | 95% C.I. | |
|  |  |  |  | lower | upper |
| PT | 0.589 | 0.024 | <0.001 | 0.542 | 0.637 |
| Years of work | 0.582 | 0.027 | 0.003 | 0.528 | 0.635 |

**Table 3:** Results of ROC curves of prevention strategies for work-related injuries.

| **Area Under Curve (AUC)** | | | | | |
| --- | --- | --- | --- | --- | --- |
| Test variable | AUC | standant error^a^ | Progressive Sig.^b^ | 95% C.I. | |
|  |  |  |  | lower | upper |
| Utilizing equipment | 0.563 | 0.021 | 0.003 | 0.522 | 0.604 |
| Improving technology | 0.587 | 0.021 | <0.001 | 0.547 | 0.628 |
| Patient engagement | 0.601 | 0.017 | <0.001 | 0.567 | 0.635 |
| Relevant education | 0.611 | 0.022 | <0.001 | 0.568 | 0.654 |
| Habit of regular exercise | 0.573 | 0.021 | <0.001 | 0.531 | 0.616 |
